# Clinical course of choroidal neovascular membrane in West Nile virus chorioretinitis: a case report

**DOI:** 10.1186/s13256-021-02700-0

**Published:** 2021-04-19

**Authors:** Roberta Zito, Tommaso Micelli Ferrari, Luigi Di Pilato, Massimo Lorusso, Anna Ferretta, Luisa Micelli Ferrari, Massimo Accorinti

**Affiliations:** 1 Department of Ophthalmology, Hospital “F. Miulli”, Acquaviva delle Fonti, 70124 Bari, Italy; 2 Department of Basic Medical Sciences, Neurosciences and Sensory Organs, University of Bari “Aldo Moro”, 70124 Bari, Italy; 3 Department of Ophthalmology, Azienda Ospedaliero-Universitaria, Policlinico, Bari, Italy; 4 Department of Ophthalmology, Ocular Immunovirology Service, Sapienza University of Rome, Rome, Italy

**Keywords:** Chorioretinitis, WNV, Retinal vasculitis, Bevacizumab

## Abstract

**Background:**

This report describes the clinical course of choroidal neovascular membrane (CNV) in West Nile virus-associated chorioretinitis.

**Case presentation:**

A 28-year-old Italian woman was referred to our institution because of reduced visual acuity in the left eye dating back 4 months. A diagnosis of retinal vasculitis in the right eye and chorioretinitis with CNV in the left eye was made. A complete workup for uveitis revealed positivity only for anti-West Nile virus immunoglobulin M (IgM), while immunoglobulin G (IgG) was negative. Whole-body computed tomography and nuclear magnetic resonance imaging of the brain were also negative. Therefore, the patient was treated with a combination of oral prednisone (starting dose 1 mg/kg per day) and three intravitreal injections of bevacizumab 1.25 mg/0.05 ml, 1 month apart. Fourteen days from starting corticosteroid therapy and after the first intravitreal injection, the patient experienced increased visual acuity to 0.4. Response to therapy was monitored by clinical examination, ocular coherence tomography (OCT), OCT angiography and retinal fluorescein angiography. Three months later, resolution of CNV in the left eye was achieved and no signs of retinal vasculitis were detected in the right eye, while serum IgM for West Nile virus turned negative and IgG positive.

**Conclusion:**

CNV may be a complication of West Nile virus-associated chorioretinitis, and only subclinical retinal vasculitis may also be found even in non-endemic regions.

## Background

West Nile virus (WNV) is a single-stranded ribonucleic acid (RNA) virus that belongs to the family *Flaviviridae*. It causes a zoonosis transmitted from birds to humans through mosquitos. This infectious disease has spread rapidly in America since 2015, but it has also been reported as endemic in Europe, Asia and Africa. WNV infection is quite rare in Italy.

Clinical manifestations of WNV infection can occur as an asymptomatic form (nearly 80% of cases), as West Nile fever (20% of subjects) and as West Nile meningoencephalitis in less than 1%. This last clinical manifestation is mainly related to immunosuppression or diabetes. The WNV incubation period is 3 to 14 days, after which fever, headache, diffuse myalgia, asthenia, gastrointestinal symptoms and maculopapular rash may occur. Neurological involvement includes meningitis, encephalitis and meningoencephalitis [1]. Treatment consists of intravenous injection of alpha interferon, ribavirin and immunoglobulins, which have been proposed only for the most severe forms.

Several ocular manifestations of WNV infection, including occlusive ischemic stroke, optic neuritis, sixth cranial nerve palsy, nystagmus, multifocal chorioretinitis, chorioretinal scarring and uveitis without focal lesions, have been described. The most common manifestation is chorioretinitis characterized by the presence of 100–1500-micron circular lesions with cream-colored margins in the active form or well-defined chorioretinal scars after resolution. Sometimes a mild to moderate vitreous haze can be found in the active form [2].

Fluorescein angiography (FAG) and indocyanine green angiography (ICGA) are essential ancillary tools used in WNV-associated ocular involvement. Active lesions, which are deep and cream-colored on fundus examination, during the early phase of FAG are round-shaped hyperfluorescent dots with subsequent late staining. Inactive lesions are atrophic and partially pigmented, centrally hypofluorescent with some hyperfluorescence at the margins. At ICGA, lesions appear hypocyanescent. Ocular coherence tomography (OCT) scan shows deep lesions not involving the internal retinal layers and the nerve fiber layer [3].

Diagnosis of WNV-associated ocular lesions is based on clinical examination, detection of specific immunoglobulin M (IgM) antibodies and exclusion of other more common forms of uveitis [4]. Excluding the most severe cases, for which hospitalization is needed, West Nile chorioretinitis is considered to be a self-limiting pathology with a partial recovery of visual function if secondary inflammatory choroidal neovascularization (CNV) does not occur [5].

The aim of this case report is to describe, in the first case of WNV-associated chorioretinitis diagnosed in Italy, the clinical course of the disease and its complications.

## Case presentation

A 28-year-old Italian woman was referred to our institution because of reduced visual acuity in the left eye dating back 4 months.

**Fig. 1:**
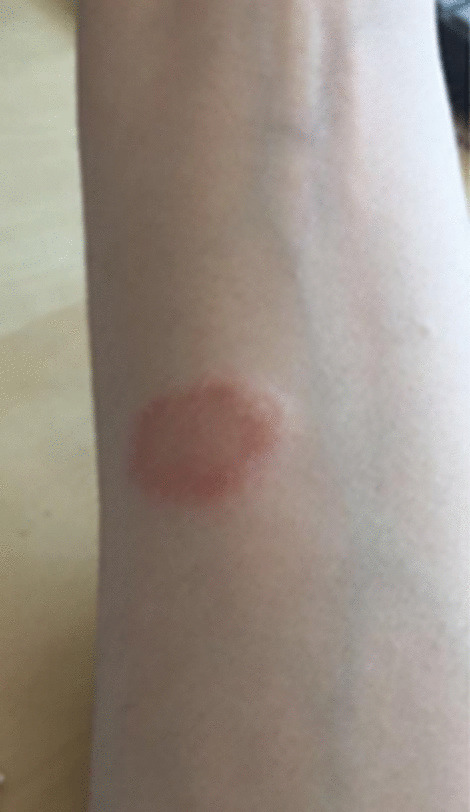
Urticaria-like reaction diagnosed as contact dermatitis. The patient developed persistent fever (37.5 °C) for about 1 month, occasionally accompanied by urticaria-like reactions on her trunk and limbs

Medical history was unremarkable from 4–5 months prior to our observation, when the patient developed persistent fever (37.5°C) for about 1 month, occasionally accompanied by urticaria-like reactions on her trunk and limbs, diagnosed as contact dermatitis (Fig. 1).

At that time the patient was living in the northern part of Italy, working at a company producing poultry feed and soil fertilizers. Two weeks after the onset of systemic lesions, she developed a sudden drop in visual acuity in her left eye. She was diagnosed elsewhere with myopia-related CNV and was treated with two intravitreal injections of ranibizumab 0.5 mg, 1 month apart, without any improvement.

## Clinical findings

**Fig. 2:**
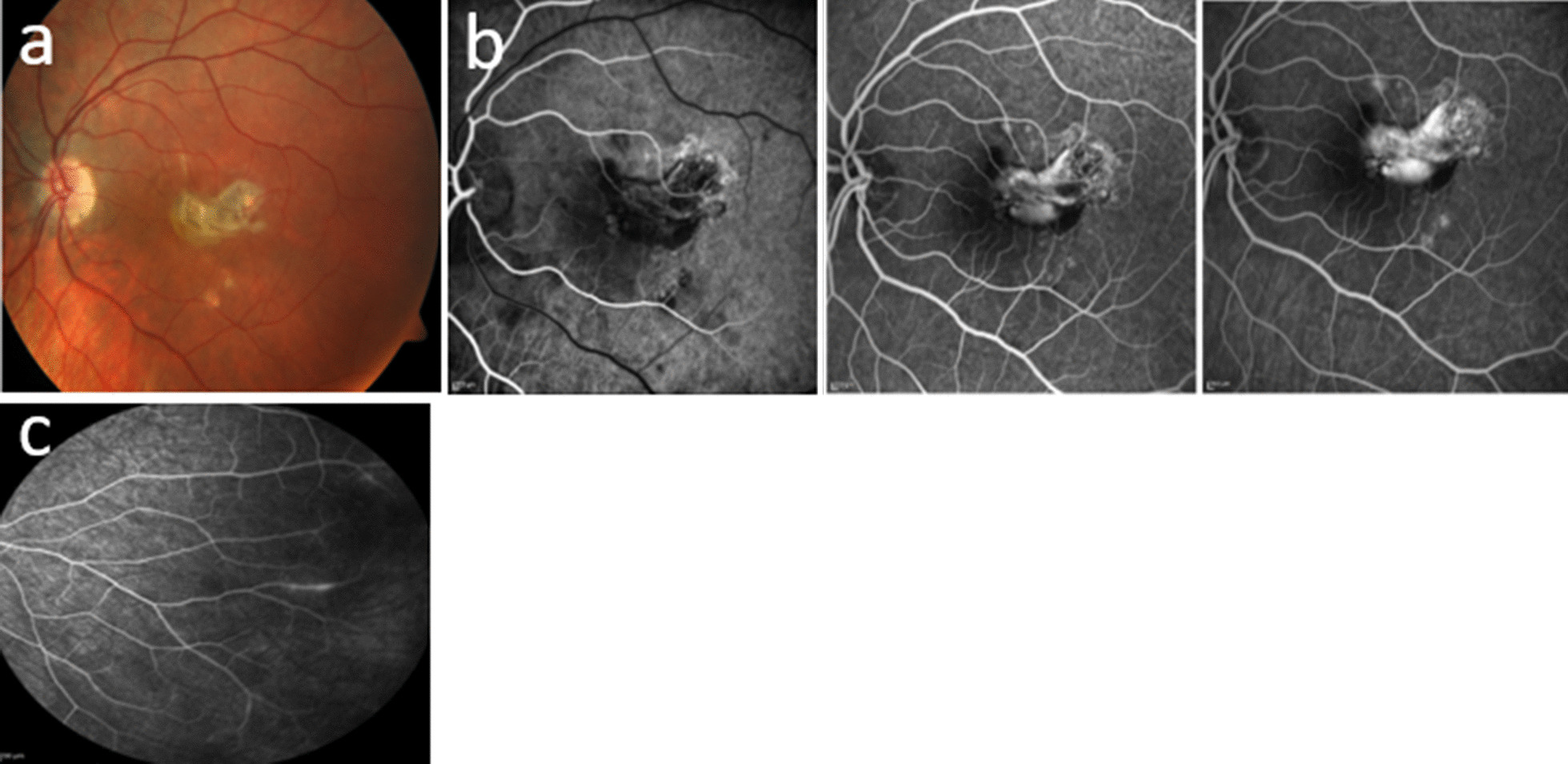
Baseline.: **a** Left eye retinography: partially pigmented ameboid yellowish macular lesion and small, round, cream-colored spots in the perimacular area. **b** Left eye fluorescein angiography: active choroidal macular neovascularization and hyperfluorescent spots near the fovea with no modification over time (atrophic changes). **c** Right eye fluorescein angiography: leakage in the periphery

During our first examination, she presented with mild myopia (−1.5 diopters) and best-corrected visual acuity (BCVA) of 0.2 in the left eye, normal anterior segment, no cataract, and a partially pigmented yellowish ameboid lesion in the macular region. Small, round, cream-colored spots were observed in the perimacular region (Fig. 2a). BCVA in the right eye was 1 (−2 diopters). Anterior and posterior segments were both normal. Intraocular pressure was 14 mmHg in both eyes.

## Diagnostic assessment

**Fig. 3:**
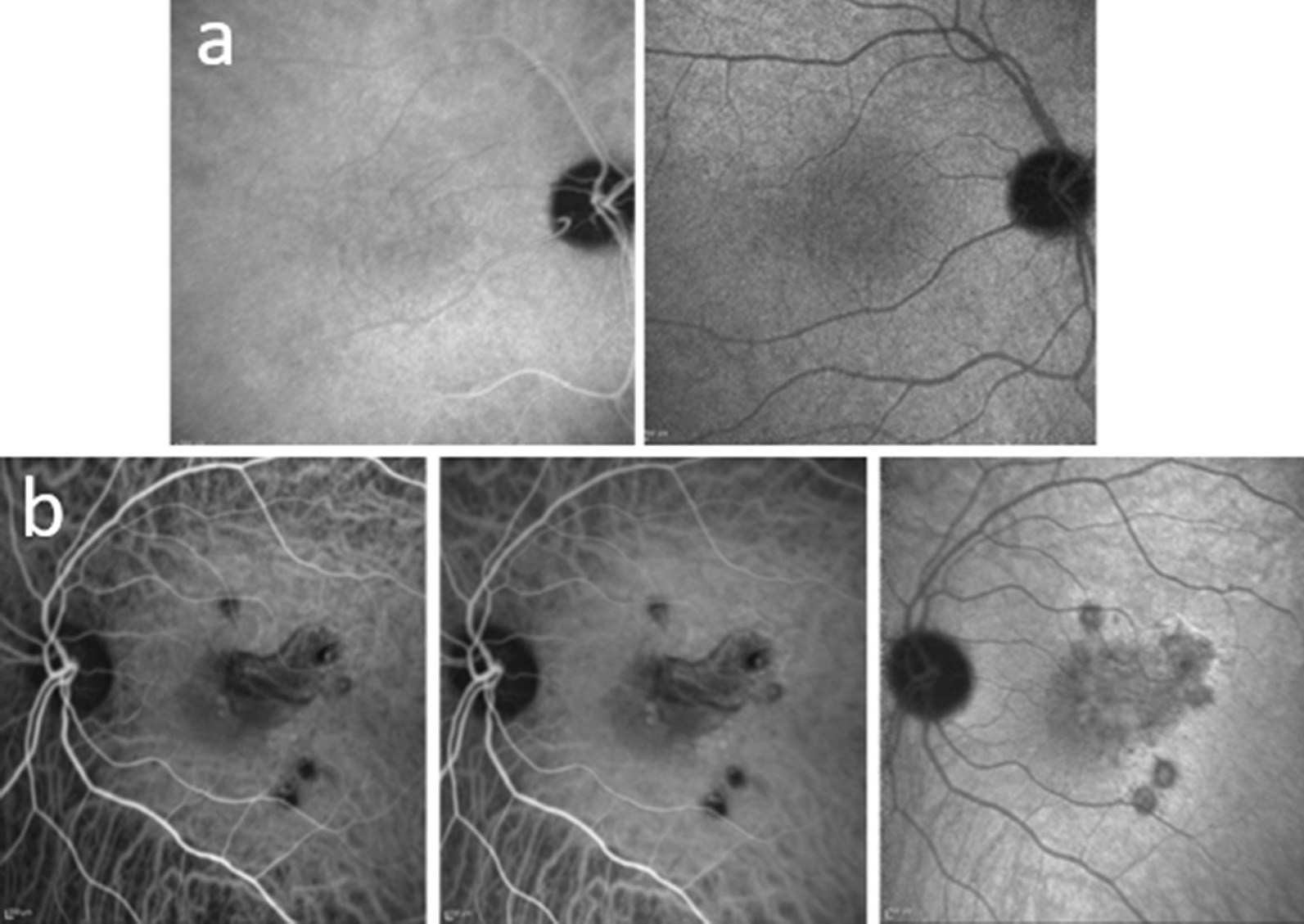
Indocyanine green angiography. **a** Right eye: normal. **b** Left eye: hypocyanescent annular rim of the perimacular lesions, with mild central hypercyanescence

Multimodal imaging was carried out and demonstrated active retinal vasculitis in the right eye (Figs. 2c, 3a) (undetectable on ophthalmoscopy) and active CNV in combination with multiple chorioretinal scars in the left eye (Figs. 2a, b, 3b).

The clinical history of the patient, the signs of retinal vasculitis in the contralateral eye and the mild myopia suggested an investigation of possible infectious retinal disease as differential diagnosis.

Therefore, we performed a complete workup for uveitis including Mantoux skin test and QuantiFERON-TB Gold test, Venereal Disease Research Laboratory (VDRL) and Treponema pallidum particle agglutination (TPHA) tests, anti-*Toxoplasma* and anti-*Toxocara* antibodies, antiviral antibodies (Herpes simplex virus type-1 [HSV1], Herpes simplex virus type-2 [HSV2], Epstein-Barr virus [EBV], Cytomegalovirus [CMV], Varicella-zoster virus [VZV]), anti-*Brucella*, anti-*Bartonella* and anti-*Rickettsia* antibodies and blood cell count, along with erythrocyte sedimentation rate, C-reactive protein, and liver and kidney evaluation tests. Furthermore, considering the latest news reporting some cases of West Nile in the northern part of Italy, where the patient had spent some months, we also investigated for West Nile IgM and IgG antibodies. The laboratory results showed an increase in lymphocytes, erythrocyte sedimentation rate and C-reactive protein, and positivity for anti-West Nile virus IgM, while IgG was negative.

Hence, the patient was sent to an infectious diseases specialist and underwent a whole-body computed tomography scan and nuclear magnetic resonance imaging of the brain, which both yielded normal results. Therefore, a minor self-limiting West Nile infection with ocular involvement complicated by CNV was diagnosed.

## Therapeutic intervention

The patient was treated with a combination of oral corticosteroids (starting dose: 1 mg/kg per day of prednisone, slowly tapered and stopped in 12 weeks) and three intravitreal injections of bevacizumab 1.25 mg/0.05 ml, 1 month apart, because ranibizumab is not approved in Italy for post-inflammatory choroidal neovascularization. Fourteen days from starting corticosteroid therapy and after the first intravitreal injection, the patient experienced an increase in BCVA to 0.4.

## Follow-up and outcomes

**Fig. 4:**
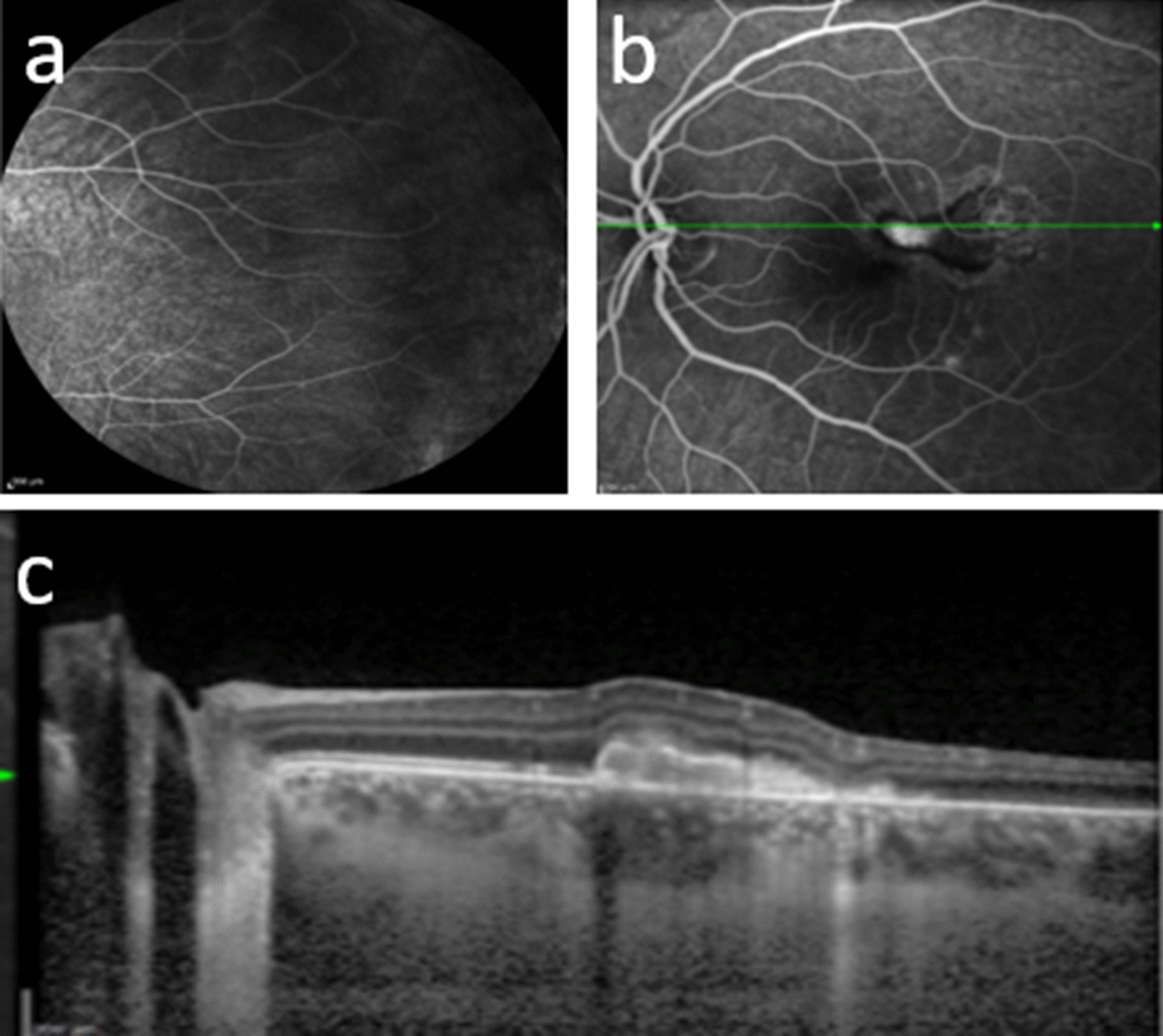
Three months after starting therapy. **a** Fluorescein angiography, right eye: complete resolution of the vascular leakage. **b** Fluorescein angiography, left eye: significant decrease in macular hyperfluorescence. **c** Ocular coherence tomography, left eye: decreased volume of the hyperreflective dome-shaped lesion with atrophic and fibrotic changes

Response to therapy was monitored by clinical examination, OCT, OCT angiography and retinal FAG. ICGA was not repeated because of fainting occurring during the first exam. Clinical findings 3 months after starting therapy are shown in Fig. 4: resolution of CNV was achieved, with no intraretinal fluid in the left eye, while no vasculitis was detected in the right eye; serum IgM for WNV turned negative and IgG positive.

After stopping therapy, the patient was followed up for an additional 14 months, with no evidence of recurrence of active inflammation or choroidal neovascularization. Final BCVA was 0.5 in the left eye and 1 in the right eye.

## Discussion

WNV infection is an emerging disease worldwide, and ocular involvement may occur as an asymptomatic form or as lesions with significant morbidity [1].

Systemic therapy for WNV infection is usually suggested by infectious disease specialists to treat and control only the most severe cases, namely those with neurologic involvement. Nevertheless, our case demonstrated that systemic therapy with corticosteroids may be beneficial for the treatment of subclinical retinal lesions, such as retinal vasculitis. There were no retinal lesions in the eye presenting with retinal vasculitis. This can be found only with FAG and might occur even independently from the presence of chorioretinal lesions or scars. In such a case, systemic antiviral therapy was not needed, because systemic active lesions were absent.

Chorioretinal lesions are the most frequent inflammatory ocular findings, but they do not present any peculiar features. Choroidal neovascularization may occur as a complication in patients with chorioretinal lesions of different etiologies, with elapsed time from the acute disease ranging from weeks to years [6].

In those cases, intravitreal injections of anti-vascular endothelial growth factor (VEGF) are a reasonable approach, leading to resolution of the lesions usually with only a few intravitreal injections.

**Fig. 5:**
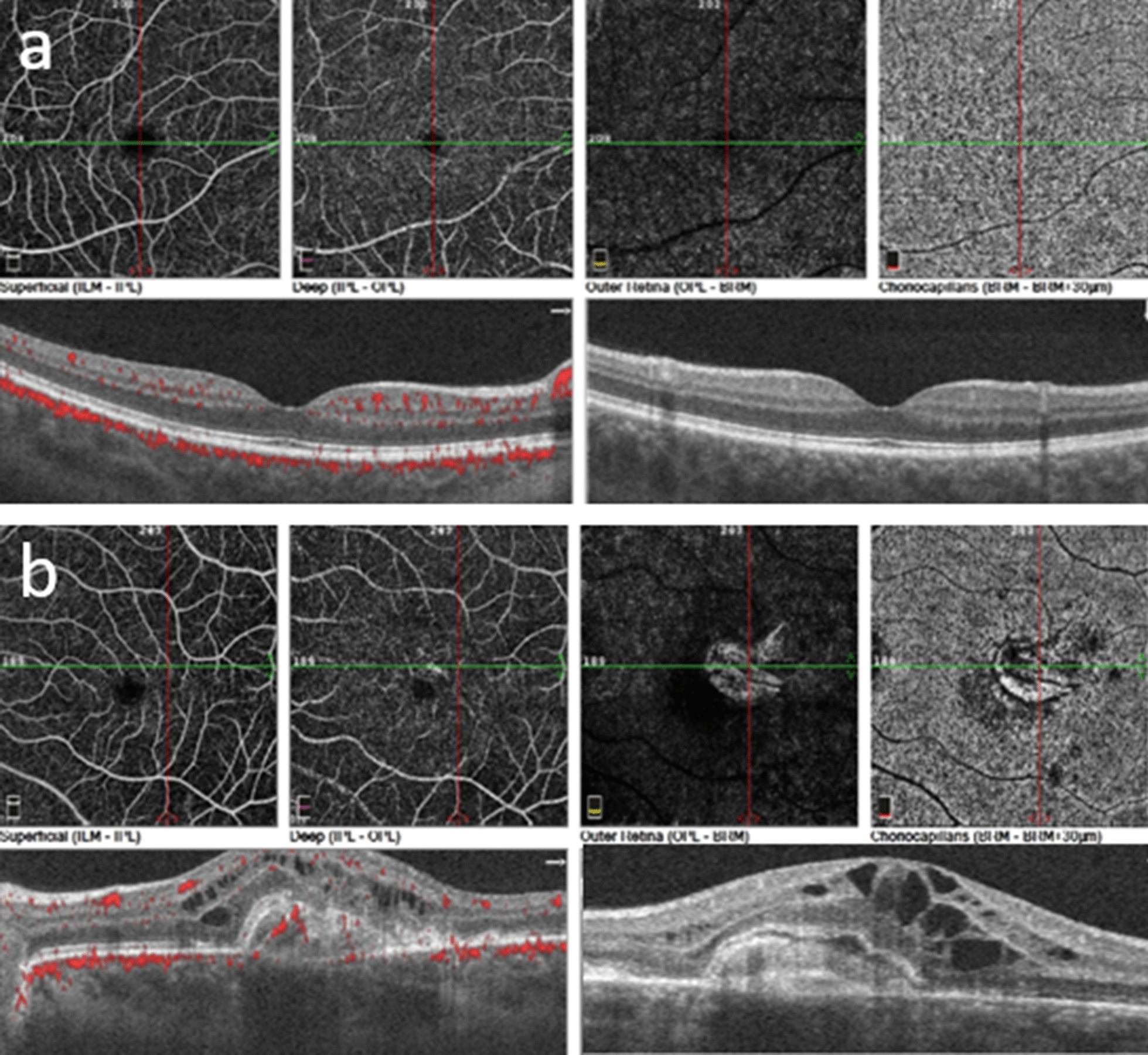
Optical coherence tomography (OCT) and OCT angiography. **a** Right eye: normal. **b** Left eye: neovascularization at the choriocapillaris level (3 × 3 and 6 × 6 images) and changes in the macular profile, increased central retinal thickness, and large hyperreflective domed lesion starting from the RPE-CC. Large cystic intraretinal spaces mainly located in the inner nuclear layer

No significant difference in terms of ocular recovery has been demonstrated among different anti-VEGF drugs. In our patient, diagnosis of WNV infection was made based on the patient’s history of positivity to the specific serologic tests. Nevertheless, any test useful for excluding other more common entities was done and resulted in negative findings. CNV was the first symptomatic lesion recalled by the patient, and it failed to achieve any significant results with two injections of ranibizumab. Therefore, considering the presence of active vasculitis in the contralateral eye and the active CNV, we decided to treat the patient with three further injections of bevacizumab, given in combination with systemic corticosteroid therapy. This therapeutic regimen enabled complete resolution of the neovascularization and retinal vasculitis (Fig. 4a), with a relapse-free period of 14 months after therapy withdrawal. It is possible that a higher number of injections and, probably, the combined treatment with systemic corticosteroid and anti-VEGF intravitreal injections, as suggested by other authors, led to the resolution of CNV in our patient. OCT and OCT angiography were essential tools in the follow-up of the complications found in our case (Fig. 5): they are noninvasive, and in a patient with suspected intolerance to ICGA, they were able to adequately follow the clinical course of both CNV and chorioretinal lesions. Nevertheless, as previously suggested, they should be performed, if possible, in combination with FAG and ICGA, which are able to detect subclinical inflammation in retinal vessels and in the choroid. In conclusion, WNV ocular infection is another ocular inflammatory disease in which a multimodal imaging approach is essential for adequate diagnosis and follow-up of the disease course and its response to treatment.

## Conclusions

Our case highlights the possibility that even in a non-endemic country, WNV may occur, along with its ocular manifestations, namely CNV. Not all ocular lesions are symptomatic: vasculitis may occur in a chorioretinal lesion-free eye, and even the presence of chorioretinitis may be asymptomatic [7]. Nevertheless, chorioretinal asymptomatic lesions may become symptomatic after the development of chorioretinal neovascularization. A viral infection can also lead to unilateral involvement, for example, in CMV infection and in patients suffering from herpes-related acute retinal necrosis.

*Patient perspective*: In such a case, combined therapy with systemic corticosteroids and anti-VEGF may control all the ocular manifestations, without recurrence over medium-term follow-up.

## Data Availability

The data sets used and/or analyzed during the current study are available from the corresponding author on reasonable request.

## Abbreviations

BCVA: Best-corrected visual acuity
CNV: Choroidal neovascular membrane
FAG: Fluorescein angiography
ICGA: Indocyanine green angiography
OCT: Ocular coherence tomography
WNV: West Nile virus
VEGF: Vascular endothelial growth factor
